# (Acetyl­acetonato)dibromido[2,2-diphenyl­hydrazin-1-ido(1−)][2,2-diphenyl­hydrazin-1-ido(2−)]molybdenum(VI)

**DOI:** 10.1107/S1600536811015881

**Published:** 2011-05-07

**Authors:** Carlos Bustos, Luis Alvarez-Thon, Andrés Ibañez, Christian Sánchez

**Affiliations:** a Instituto de Ciencias Químicas, Universidad Austral de Chile, Avda. Los Robles s/n, Campus Isla Teja, Casilla 567, Valdivia, Chile; b Departamento de Ciencias Físicas, Universidad Andres Bello, Avda. República 220, Santiago de Chile, Chile; c Laboratorio de Cristalografía, Difracción de Rayos-X, Departamento de Física, Facultad de Ciencias Físicas y Matemáticas, Universidad de Chile, Av. Blanco Encalada 2008, Santiago, Chile

## Abstract

In the title compound, [MoBr_2_(C_12_H_11_N_2_)(C_12_H_10_N_2_)(C_5_H_7_O_2_)], the Mo^VI^ atom is six-coordinated in a distorted octa­hedral geometry by two N atoms from the diphenyl­hydrazide(1−) and diphenyl­hydrazide(2−) ligands, two O atoms from a bidentate acetyl­acetonate ligand and two Br^−^ ions. The mol­ecules form an inversion dimer *via* a pair of weak C—H⋯O hydrogen bonds and a π–π stacking inter­action with a centroid–centroid distance of 3.7401 (12) Å. Weak intra­molecular C—H⋯Br inter­actions and an intra­molecular π–π stacking inter­action with a centroid–centroid distance of 3.8118 (15) Å are also observed.

## Related literature

For related structures, see: Bustos *et al.* (1994 ▶, 2006 ▶). For the importance of these compounds as potential models of inter­mediates in the conversion of coordinated dinitro­gen into ammonia, see: Henderson *et al.* (1983 ▶); McCleverty (1987 ▶).
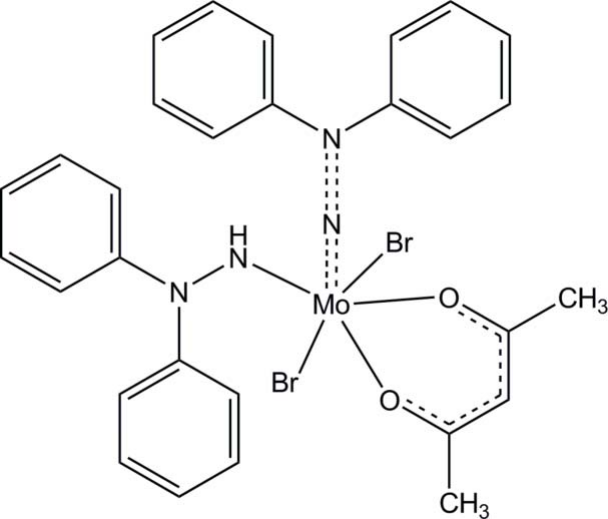


## Experimental

### 

#### Crystal data

[MoBr_2_(C_12_H_11_N_2_)(C_12_H_10_N_2_)(C_5_H_7_O_2_)]
*M* *_r_* = 720.29
Monoclinic, 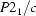

*a* = 9.5828 (11) Å
*b* = 32.187 (4) Å
*c* = 9.1455 (10) Å
β = 94.601 (2)°
*V* = 2811.8 (6) Å^3^
*Z* = 4
Mo *K*α radiation
μ = 3.34 mm^−1^
*T* = 150 K
0.36 × 0.31 × 0.29 mm

#### Data collection

Bruker D8 Discover with SMART CCD area-detector diffractometer
Absorption correction: multi-scan (*SADABS*; Bruker, 2000 ▶) *T*_min_ = 0.324, *T*_max_ = 0.379
22331 measured reflections
5678 independent reflections
5133 reflections with *I* > 2σ(*I*)
*R*_int_ = 0.021

#### Refinement

*R*[*F* ^2^ > 2σ(*F* ^2^)] = 0.024
*wR*(*F* ^2^) = 0.063
*S* = 1.05
5678 reflections
349 parameters
H atoms treated by a mixture of independent and constrained refinement
Δρ_max_ = 0.68 e Å^−3^
Δρ_min_ = −0.29 e Å^−3^

### 

Data collection: *SMART* (Bruker, 2001 ▶); cell refinement: *SAINT* (Bruker, 2000 ▶); data reduction: *SAINT*; program(s) used to solve structure: *SHELXS97* (Sheldrick, 2008 ▶); program(s) used to refine structure: *SHELXL97* (Sheldrick, 2008 ▶); molecular graphics: *SHELXTL* (Sheldrick, 2008 ▶); software used to prepare material for publication: *PLATON* (Spek, 2009 ▶) and *Mercury* (Macrae *et al.*, 2006 ▶).

## Supplementary Material

Crystal structure: contains datablocks global, I. DOI: 10.1107/S1600536811015881/is2705sup1.cif

Structure factors: contains datablocks I. DOI: 10.1107/S1600536811015881/is2705Isup2.hkl

Additional supplementary materials:  crystallographic information; 3D view; checkCIF report

## Figures and Tables

**Table 1:** Selected bond lengths (Å)

Mo1—Br1	2.6023 (5)
Mo1—Br2	2.5646 (4)
Mo1—O1	2.1074 (16)
Mo1—O2	2.0530 (13)
Mo1—N1	1.9638 (16)
Mo1—N3	1.7559 (18)

**Table 2:** Hydrogen-bond geometry (Å, °)

*D*—H⋯*A*	*D*—H	H⋯*A*	*D*⋯*A*	*D*—H⋯*A*
C6—H6⋯Br2	0.95	2.92	3.712 (3)	142
C20—H20⋯Br2	0.95	2.85	3.788 (2)	168
C23—H23⋯O2^i^	0.95	2.49	3.392 (2)	158

Symmetry code: (i) 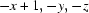.

**Fig. 1.**
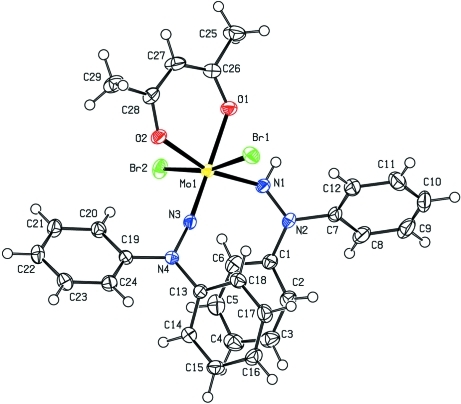
View of the title compound with the atom numbering scheme. Displacement ellipsoids for non-H atoms are drawn at the 50% probability level.

**Fig. 2.**
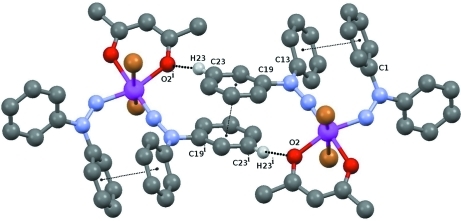
Part of the crystal structure, showing the formation of dimer connected via two weak contacts (dashed lines) and one π–π stacking interaction. It is also shown intramolecular π–π stacking interactions (dotted lines) [symmetry code: (i) 1 - x, -y, -z]. H atoms not involved in the interactions have been omitted for clarity.

## supplementary crystallographic information

### Comment

In a previous paper, it was published a series of molybdenum complexes containing both end-on organohydrazide(1-) and organohydrazide(2-) ligands (Bustos *et al.*, 1994), formulated as [Mo(NHNRPh)(NNRPh)(acac)*X*_2_] (*R*= Me, Ph; *X* = Cl, Br, I). These compounds are of remarkable importance as potential models of intermediates in the conversion of coordinated dinitrogen into ammonia (McCleverty, 1987; Henderson *et al.*, 1983). Here, the crystalline and molecular structure of [Mo(NHNPh_2_)(NNPh_2_)(acac)Br_2_] is reported.

The molecular structure of the title compound, (I), is shown in Fig. 1. This compound which crystallizes in space group *P*2_1_/*c*, is equivalent in topology to [Mo(NHNPh_2_)(NNPh_2_)(acac)Cl_2_] (Bustos *et al.*, 1994), (code HEDCIC), where the bromine atoms are replaced by chlorine atoms.

The coordination geometry about the Mo^VI^ atom can be described as a distorted octahedron (Table 1). The bromine ligands occupy two *trans*-axial sites while the equatorial positions are occupied by two *cis*-hydrazide ligands and the oxygen atoms of the acetylacetonate ligand. The two hydrazide ligands are different due to the location of the hydrogen atom attached to the N1 atom. In effect the Mo1, N3 and N4 atoms are almost linear [171.87 (14)°] while the Mo1—N1—N2 angle is 140.98 (15)°. In (I), there are two weak intramolecular C—H···Br hydrogen bonds (Table 2) and one intramolecular π–π stacking interaction involving the C1—C6 and C13—C18 rings, with a distance of 3.8118 (15) Å between ring centroids (Fig. 2).

In the crystal structure, two weak hydrogen bonds C23—H23···O2^i^ and C23^i^—H23^i^..O2 (Table 2) link the molecules into dimers. In addition these dimers are further stabilized by a π–π stacking interaction with distance *Cg*···*Cg*^i^ of 3.7401 (12) Å, where *Cg* is the centroids of the C19–C24 ring (Fig. 2). [symmetry code: (i) 1 - *x*, -*y*, -*z*]. The entire three-dimensional network is constructed mainly by weak C—H···Br interactions (Bustos *et al.*, 2006).

### Experimental

This compound was synthesized as was described in the literature (Bustos *et al.*, 1994), and single crystals suitable for X-ray diffraction were obtained by diffusion of diethylether on a concentrated solution of the compound in chloroform.

### Refinement

The H atom attached to the N1 atom was located in a difference Fourier map and refined freely. Other H atoms were placed in geometrically idealized positions and constrained to ride on their parent atoms, with aromatic C—H = 0.95 Å and *U*_iso_(H) = 1.2*U*_eq_(C), and methyl C—H = 0.98 Å and *U*_iso_(H) = 1.5*U*_eq_(C). The methyl groups were allowed to rotate.

### Crystal data

**Table:**

[MoBr_2_(C_12_H_11_N_2_)(C_12_H_10_N_2_)(C_5_H_7_O_2_)]	*F*(000) = 1432
*M**_r_* = 720.29	*D*_x_ = 1.702 Mg m^−^^3^
Monoclinic, *P*2_1_/*c*	Mo *K*α radiation, λ = 0.71073 Å
Hall symbol: -P 2ybc	Cell parameters from 999 reflections
*a* = 9.5828 (11) Å	θ = 2.1–26.3°
*b* = 32.187 (4) Å	µ = 3.34 mm^−^^1^
*c* = 9.1455 (10) Å	*T* = 150 K
β = 94.601 (2)°	Block, red
*V* = 2811.8 (6) Å^3^	0.36 × 0.31 × 0.29 mm
*Z* = 4	

### Data collection

**Table:**

Bruker D8 Discover with SMART CCD area-detector diffractometer	5678 independent reflections
Radiation source: fine-focus sealed tube	5133 reflections with *I* > 2σ(*I*)
graphite	*R*_int_ = 0.021
φ and ω scans	θ_max_ = 26.3°, θ_min_ = 2.1°
Absorption correction: multi-scan (*SADABS*; Bruker, 2000)	*h* = −11→11
*T*_min_ = 0.324, *T*_max_ = 0.379	*k* = −39→40
22331 measured reflections	*l* = −11→11

### Refinement

**Table:**

Refinement on *F*^2^	Primary atom site location: structure-invariant direct methods
Least-squares matrix: full	Secondary atom site location: difference Fourier map
*R*[*F*^2^ > 2σ(*F*^2^)] = 0.024	Hydrogen site location: inferred from neighbouring sites
*wR*(*F*^2^) = 0.063	H atoms treated by a mixture of independent and constrained refinement
*S* = 1.05	*w* = 1/[σ^2^(*F*_o_^2^) + (0.034*P*)^2^ + 1.394*P*] where *P* = (*F*_o_^2^ + 2*F*_c_^2^)/3
5678 reflections	(Δ/σ)_max_ = 0.001
349 parameters	Δρ_max_ = 0.68 e Å^−^^3^
0 restraints	Δρ_min_ = −0.29 e Å^−^^3^

### Special details

**Table:**

Geometry. Bond distances, angles *etc*. have been calculated using the rounded fractional coordinates. All su's are estimated from the variances of the (full) variance-covariance matrix. The cell e.s.d.'s are taken into account in the estimation of distances, angles and torsion angles
Refinement. Refinement on *F*^2^ for ALL reflections except those flagged by the user for potential systematic errors. Weighted *R*-factors *wR* and all goodnesses of fit *S* are based on *F*^2^, conventional *R*-factors *R* are based on *F*, with *F* set to zero for negative *F*^2^. The observed criterion of *F*^2^ > σ(*F*^2^) is used only for calculating -*R*-factor-obs *etc*. and is not relevant to the choice of reflections for refinement. *R*-factors based on *F*^2^ are statistically about twice as large as those based on *F*, and *R*-factors based on ALL data will be even larger.

### Fractional atomic coordinates and isotropic or equivalent isotropic displacement parameters (Å^2^)

**Table:**

	*x*	*y*	*z*	*U*_iso_*/*U*_eq_	
Mo1	0.81628 (2)	0.10424 (1)	0.25802 (2)	0.0187 (1)	
Br1	0.92515 (2)	0.10449 (1)	0.00633 (3)	0.0275 (1)	
Br2	0.75285 (3)	0.09355 (1)	0.52256 (2)	0.0295 (1)	
O1	1.02455 (16)	0.10749 (5)	0.35070 (18)	0.0265 (5)	
O2	0.85400 (15)	0.04148 (4)	0.25378 (16)	0.0246 (4)	
N1	0.8420 (2)	0.16473 (5)	0.26814 (19)	0.0209 (5)	
N2	0.77411 (19)	0.20059 (5)	0.2310 (2)	0.0244 (5)	
N3	0.64377 (18)	0.10063 (5)	0.17844 (19)	0.0190 (5)	
N4	0.51707 (18)	0.09218 (5)	0.11958 (19)	0.0213 (5)	
C1	0.6275 (2)	0.20319 (6)	0.2423 (2)	0.0225 (6)	
C2	0.5436 (2)	0.22312 (7)	0.1319 (3)	0.0271 (7)	
C3	0.4013 (3)	0.22633 (7)	0.1446 (3)	0.0317 (7)	
C4	0.3422 (3)	0.20957 (8)	0.2650 (3)	0.0343 (8)	
C5	0.4264 (3)	0.19004 (8)	0.3739 (3)	0.0326 (8)	
C6	0.5696 (3)	0.18676 (7)	0.3642 (3)	0.0278 (7)	
C7	0.8529 (2)	0.23412 (7)	0.1783 (3)	0.0259 (7)	
C8	0.8250 (3)	0.27458 (7)	0.2205 (3)	0.0359 (8)	
C9	0.9034 (3)	0.30687 (8)	0.1696 (4)	0.0496 (10)	
C10	1.0081 (3)	0.29879 (9)	0.0774 (4)	0.0544 (10)	
C11	1.0352 (3)	0.25879 (10)	0.0369 (3)	0.0467 (10)	
C12	0.9578 (2)	0.22561 (8)	0.0865 (3)	0.0324 (8)	
C13	0.4572 (2)	0.11994 (6)	0.0079 (2)	0.0208 (6)	
C14	0.3143 (2)	0.12864 (7)	0.0017 (2)	0.0248 (6)	
C15	0.2557 (2)	0.15429 (7)	−0.1079 (3)	0.0272 (7)	
C16	0.3388 (2)	0.17196 (7)	−0.2082 (3)	0.0296 (7)	
C17	0.4815 (2)	0.16334 (8)	−0.2006 (3)	0.0299 (7)	
C18	0.5410 (2)	0.13723 (7)	−0.0931 (2)	0.0252 (7)	
C19	0.4587 (2)	0.05174 (6)	0.1439 (2)	0.0204 (6)	
C20	0.5015 (2)	0.03015 (7)	0.2715 (2)	0.0238 (6)	
C21	0.4490 (2)	−0.00941 (7)	0.2924 (2)	0.0274 (7)	
C22	0.3528 (2)	−0.02705 (7)	0.1882 (2)	0.0262 (7)	
C23	0.3110 (2)	−0.00517 (7)	0.0626 (2)	0.0261 (7)	
C24	0.3641 (2)	0.03414 (7)	0.0386 (2)	0.0241 (6)	
C25	1.2505 (3)	0.09211 (8)	0.4574 (3)	0.0380 (8)	
C26	1.1132 (2)	0.07829 (7)	0.3829 (2)	0.0259 (7)	
C27	1.0882 (2)	0.03647 (7)	0.3542 (3)	0.0315 (7)	
C28	0.9644 (2)	0.01997 (7)	0.2893 (2)	0.0257 (7)	
C29	0.9515 (3)	−0.02542 (7)	0.2557 (3)	0.0345 (8)	
H1	0.928 (3)	0.1690 (9)	0.292 (3)	0.039 (8)*	
H2	0.58380	0.23430	0.04890	0.0320*	
H3	0.34360	0.24010	0.07040	0.0380*	
H4	0.24410	0.21150	0.27250	0.0410*	
H5	0.38560	0.17870	0.45640	0.0390*	
H6	0.62720	0.17350	0.43980	0.0330*	
H8	0.75300	0.28000	0.28360	0.0430*	
H9	0.88540	0.33460	0.19790	0.0590*	
H10	1.06120	0.32110	0.04210	0.0650*	
H11	1.10770	0.25360	−0.02570	0.0560*	
H12	0.97640	0.19790	0.05800	0.0390*	
H14	0.25780	0.11710	0.07210	0.0300*	
H15	0.15800	0.15980	−0.11440	0.0330*	
H16	0.29840	0.19000	−0.28230	0.0360*	
H17	0.53830	0.17550	−0.26960	0.0360*	
H18	0.63830	0.13120	−0.08840	0.0300*	
H20	0.56590	0.04230	0.34340	0.0290*	
H21	0.47890	−0.02460	0.37830	0.0330*	
H22	0.31610	−0.05400	0.20360	0.0310*	
H23	0.24510	−0.01710	−0.00840	0.0310*	
H24	0.33610	0.04890	−0.04890	0.0290*	
H25A	1.29850	0.11010	0.39110	0.0570*	
H25B	1.30890	0.06780	0.48280	0.0570*	
H25C	1.23390	0.10750	0.54690	0.0570*	
H27	1.16190	0.01760	0.38120	0.0380*	
H29A	0.85890	−0.03530	0.27850	0.0520*	
H29B	1.02400	−0.04070	0.31510	0.0520*	
H29C	0.96300	−0.03000	0.15140	0.0520*	

### Atomic displacement parameters (Å^2^)

**Table:**

	*U*^11^	*U*^22^	*U*^33^	*U*^12^	*U*^13^	*U*^23^
Mo1	0.0180 (1)	0.0146 (1)	0.0227 (1)	−0.0005 (1)	−0.0028 (1)	0.0006 (1)
Br1	0.0241 (1)	0.0284 (1)	0.0307 (1)	−0.0020 (1)	0.0060 (1)	−0.0039 (1)
Br2	0.0391 (1)	0.0259 (1)	0.0226 (1)	−0.0065 (1)	−0.0034 (1)	0.0040 (1)
O1	0.0217 (8)	0.0211 (8)	0.0353 (9)	0.0005 (6)	−0.0063 (7)	−0.0004 (6)
O2	0.0245 (8)	0.0169 (7)	0.0311 (8)	0.0017 (6)	−0.0049 (6)	0.0005 (6)
N1	0.0207 (10)	0.0171 (9)	0.0244 (9)	0.0010 (7)	−0.0014 (8)	0.0006 (7)
N2	0.0248 (9)	0.0147 (9)	0.0333 (10)	0.0015 (7)	0.0008 (8)	0.0041 (8)
N3	0.0206 (9)	0.0154 (8)	0.0208 (9)	−0.0014 (7)	0.0010 (7)	0.0008 (7)
N4	0.0172 (9)	0.0213 (9)	0.0246 (9)	−0.0019 (7)	−0.0029 (7)	0.0043 (7)
C1	0.0234 (11)	0.0165 (10)	0.0274 (11)	0.0024 (8)	0.0018 (9)	−0.0032 (8)
C2	0.0297 (12)	0.0218 (11)	0.0294 (12)	0.0020 (9)	0.0009 (10)	0.0001 (9)
C3	0.0312 (13)	0.0257 (12)	0.0372 (13)	0.0056 (10)	−0.0034 (10)	−0.0043 (10)
C4	0.0260 (12)	0.0316 (13)	0.0457 (15)	0.0021 (10)	0.0058 (11)	−0.0102 (11)
C5	0.0375 (14)	0.0292 (13)	0.0327 (13)	−0.0021 (10)	0.0125 (11)	−0.0045 (10)
C6	0.0347 (13)	0.0228 (11)	0.0260 (11)	0.0035 (9)	0.0035 (10)	−0.0023 (9)
C7	0.0244 (11)	0.0207 (11)	0.0307 (12)	−0.0045 (8)	−0.0088 (9)	0.0070 (9)
C8	0.0364 (14)	0.0221 (12)	0.0466 (15)	0.0010 (10)	−0.0128 (11)	0.0052 (11)
C9	0.0506 (18)	0.0220 (13)	0.070 (2)	−0.0098 (12)	−0.0329 (16)	0.0139 (13)
C10	0.0444 (17)	0.0439 (17)	0.069 (2)	−0.0262 (14)	−0.0325 (16)	0.0357 (15)
C11	0.0277 (13)	0.062 (2)	0.0485 (16)	−0.0142 (13)	−0.0088 (12)	0.0283 (15)
C12	0.0289 (13)	0.0319 (13)	0.0351 (13)	−0.0045 (10)	−0.0045 (10)	0.0088 (10)
C13	0.0206 (10)	0.0181 (10)	0.0229 (10)	−0.0017 (8)	−0.0034 (8)	0.0012 (8)
C14	0.0219 (11)	0.0251 (11)	0.0275 (11)	−0.0014 (9)	0.0023 (9)	0.0023 (9)
C15	0.0211 (11)	0.0280 (12)	0.0316 (12)	0.0022 (9)	−0.0039 (9)	0.0009 (10)
C16	0.0324 (13)	0.0286 (12)	0.0265 (12)	0.0010 (10)	−0.0062 (10)	0.0067 (10)
C17	0.0289 (12)	0.0340 (13)	0.0266 (11)	−0.0079 (10)	0.0013 (10)	0.0069 (10)
C18	0.0204 (11)	0.0282 (12)	0.0266 (11)	−0.0024 (9)	−0.0014 (9)	0.0014 (9)
C19	0.0177 (10)	0.0183 (10)	0.0255 (11)	−0.0009 (8)	0.0031 (8)	−0.0001 (8)
C20	0.0236 (11)	0.0249 (11)	0.0225 (11)	−0.0035 (9)	−0.0003 (9)	0.0009 (9)
C21	0.0320 (12)	0.0248 (12)	0.0255 (11)	−0.0033 (9)	0.0024 (9)	0.0054 (9)
C22	0.0307 (12)	0.0194 (11)	0.0291 (12)	−0.0052 (9)	0.0070 (10)	−0.0011 (9)
C23	0.0257 (11)	0.0257 (12)	0.0264 (11)	−0.0064 (9)	−0.0005 (9)	−0.0048 (9)
C24	0.0251 (11)	0.0252 (11)	0.0216 (11)	−0.0016 (9)	−0.0006 (9)	0.0015 (9)
C25	0.0255 (13)	0.0363 (14)	0.0501 (16)	0.0037 (10)	−0.0107 (11)	−0.0099 (12)
C26	0.0206 (11)	0.0294 (12)	0.0273 (11)	0.0038 (9)	−0.0001 (9)	−0.0007 (9)
C27	0.0263 (12)	0.0274 (12)	0.0392 (14)	0.0106 (10)	−0.0075 (10)	−0.0061 (10)
C28	0.0311 (12)	0.0209 (11)	0.0246 (11)	0.0038 (9)	−0.0005 (9)	−0.0001 (9)
C29	0.0399 (14)	0.0213 (12)	0.0405 (14)	0.0071 (10)	−0.0082 (11)	−0.0053 (10)

### Geometric parameters (Å, °)

**Table:**

Mo1—Br1	2.6023 (5)	C20—C21	1.388 (3)
Mo1—Br2	2.5646 (4)	C21—C22	1.393 (3)
Mo1—O1	2.1074 (16)	C22—C23	1.379 (3)
Mo1—O2	2.0530 (13)	C23—C24	1.388 (3)
Mo1—N1	1.9638 (16)	C25—C26	1.500 (3)
Mo1—N3	1.7559 (18)	C26—C27	1.389 (3)
O1—C26	1.285 (3)	C27—C28	1.389 (3)
O2—C28	1.284 (2)	C28—C29	1.496 (3)
N1—N2	1.355 (2)	C2—H2	0.9500
N2—C1	1.419 (3)	C3—H3	0.9500
N2—C7	1.423 (3)	C4—H4	0.9500
N3—N4	1.316 (2)	C5—H5	0.9500
N4—C13	1.441 (3)	C6—H6	0.9500
N4—C19	1.441 (3)	C8—H8	0.9500
N1—H1	0.85 (3)	C9—H9	0.9500
C1—C6	1.389 (3)	C10—H10	0.9500
C1—C2	1.395 (3)	C11—H11	0.9500
C2—C3	1.382 (3)	C12—H12	0.9500
C3—C4	1.387 (4)	C14—H14	0.9500
C4—C5	1.382 (4)	C15—H15	0.9500
C5—C6	1.386 (4)	C16—H16	0.9500
C7—C12	1.388 (3)	C17—H17	0.9500
C7—C8	1.390 (3)	C18—H18	0.9500
C8—C9	1.385 (4)	C20—H20	0.9500
C9—C10	1.386 (5)	C21—H21	0.9500
C10—C11	1.370 (4)	C22—H22	0.9500
C11—C12	1.397 (4)	C23—H23	0.9500
C13—C14	1.394 (3)	C24—H24	0.9500
C13—C18	1.388 (3)	C25—H25A	0.9800
C14—C15	1.383 (3)	C25—H25B	0.9800
C15—C16	1.384 (3)	C25—H25C	0.9800
C16—C17	1.392 (3)	C27—H27	0.9500
C17—C18	1.381 (3)	C29—H29A	0.9800
C19—C24	1.389 (3)	C29—H29B	0.9800
C19—C20	1.392 (3)	C29—H29C	0.9800
			
Br1—Mo1—Br2	167.70 (1)	O1—C26—C25	115.3 (2)
Br1—Mo1—O1	85.51 (4)	O1—C26—C27	124.39 (18)
Br1—Mo1—O2	84.42 (4)	C26—C27—C28	125.5 (2)
Br1—Mo1—N1	88.90 (5)	C27—C28—C29	121.0 (2)
Br1—Mo1—N3	93.70 (6)	O2—C28—C27	124.1 (2)
Br2—Mo1—O1	85.17 (5)	O2—C28—C29	114.94 (19)
Br2—Mo1—O2	86.63 (4)	C1—C2—H2	120.00
Br2—Mo1—N1	97.29 (5)	C3—C2—H2	120.00
Br2—Mo1—N3	95.49 (6)	C2—C3—H3	120.00
O1—Mo1—O2	83.91 (6)	C4—C3—H3	120.00
O1—Mo1—N1	79.66 (7)	C3—C4—H4	120.00
O1—Mo1—N3	178.77 (7)	C5—C4—H4	120.00
O2—Mo1—N1	162.71 (7)	C4—C5—H5	120.00
O2—Mo1—N3	95.07 (7)	C6—C5—H5	120.00
N1—Mo1—N3	101.28 (8)	C1—C6—H6	121.00
Mo1—O1—C26	130.03 (14)	C5—C6—H6	121.00
Mo1—O2—C28	131.95 (13)	C7—C8—H8	120.00
Mo1—N1—N2	140.98 (15)	C9—C8—H8	120.00
Mo1—N3—N4	171.87 (14)	C8—C9—H9	120.00
N1—N2—C1	119.35 (16)	C10—C9—H9	120.00
N1—N2—C7	118.28 (17)	C9—C10—H10	120.00
C1—N2—C7	122.35 (16)	C11—C10—H10	120.00
N3—N4—C13	117.61 (16)	C10—C11—H11	119.00
N3—N4—C19	118.70 (16)	C12—C11—H11	120.00
C13—N4—C19	122.14 (16)	C7—C12—H12	121.00
Mo1—N1—H1	107 (2)	C11—C12—H12	121.00
N2—N1—H1	111 (2)	C13—C14—H14	120.00
N2—C1—C2	119.38 (17)	C15—C14—H14	120.00
C2—C1—C6	120.8 (2)	C14—C15—H15	120.00
N2—C1—C6	119.83 (19)	C16—C15—H15	120.00
C1—C2—C3	119.3 (2)	C15—C16—H16	120.00
C2—C3—C4	120.4 (3)	C17—C16—H16	120.00
C3—C4—C5	119.8 (3)	C16—C17—H17	120.00
C4—C5—C6	120.8 (3)	C18—C17—H17	120.00
C1—C6—C5	118.9 (2)	C13—C18—H18	120.00
C8—C7—C12	121.2 (2)	C17—C18—H18	120.00
N2—C7—C12	119.0 (2)	C19—C20—H20	120.00
N2—C7—C8	119.8 (2)	C21—C20—H20	120.00
C7—C8—C9	119.2 (3)	C20—C21—H21	120.00
C8—C9—C10	120.2 (3)	C22—C21—H21	120.00
C9—C10—C11	120.2 (3)	C21—C22—H22	120.00
C10—C11—C12	120.9 (3)	C23—C22—H22	120.00
C7—C12—C11	118.4 (2)	C22—C23—H23	120.00
N4—C13—C14	119.16 (17)	C24—C23—H23	120.00
C14—C13—C18	120.74 (18)	C19—C24—H24	120.00
N4—C13—C18	120.10 (17)	C23—C24—H24	120.00
C13—C14—C15	119.33 (18)	C26—C25—H25A	109.00
C14—C15—C16	120.26 (18)	C26—C25—H25B	110.00
C15—C16—C17	120.0 (2)	C26—C25—H25C	110.00
C16—C17—C18	120.3 (2)	H25A—C25—H25B	109.00
C13—C18—C17	119.32 (18)	H25A—C25—H25C	109.00
C20—C19—C24	120.65 (19)	H25B—C25—H25C	109.00
N4—C19—C24	120.07 (17)	C26—C27—H27	117.00
N4—C19—C20	119.25 (17)	C28—C27—H27	117.00
C19—C20—C21	119.21 (18)	C28—C29—H29A	110.00
C20—C21—C22	120.48 (18)	C28—C29—H29B	109.00
C21—C22—C23	119.6 (2)	C28—C29—H29C	109.00
C22—C23—C24	120.77 (18)	H29A—C29—H29B	109.00
C19—C24—C23	119.29 (18)	H29A—C29—H29C	109.00
C25—C26—C27	120.34 (19)	H29B—C29—H29C	110.00
			
Br1—Mo1—O1—C26	−86.31 (17)	C6—C1—C2—C3	−0.2 (3)
Br2—Mo1—O1—C26	85.66 (17)	N2—C1—C2—C3	−178.7 (2)
O2—Mo1—O1—C26	−1.46 (17)	C2—C1—C6—C5	0.7 (3)
N1—Mo1—O1—C26	−176.01 (18)	C1—C2—C3—C4	−0.6 (4)
Br1—Mo1—O2—C28	83.01 (17)	C2—C3—C4—C5	0.9 (4)
Br2—Mo1—O2—C28	−88.54 (17)	C3—C4—C5—C6	−0.3 (4)
O1—Mo1—O2—C28	−3.05 (17)	C4—C5—C6—C1	−0.5 (4)
N3—Mo1—O2—C28	176.24 (17)	N2—C7—C12—C11	−179.3 (2)
Br1—Mo1—N1—N2	90.8 (2)	N2—C7—C8—C9	179.4 (3)
Br2—Mo1—N1—N2	−99.9 (2)	C8—C7—C12—C11	−0.1 (4)
O1—Mo1—N1—N2	176.5 (2)	C12—C7—C8—C9	0.1 (4)
N3—Mo1—N1—N2	−2.7 (2)	C7—C8—C9—C10	0.2 (5)
Mo1—O1—C26—C25	−176.12 (15)	C8—C9—C10—C11	−0.4 (5)
Mo1—O1—C26—C27	3.4 (3)	C9—C10—C11—C12	0.5 (5)
Mo1—O2—C28—C27	5.7 (3)	C10—C11—C12—C7	−0.2 (4)
Mo1—O2—C28—C29	−174.32 (15)	C18—C13—C14—C15	1.0 (3)
Mo1—N1—N2—C1	36.7 (3)	N4—C13—C14—C15	−178.12 (19)
Mo1—N1—N2—C7	−141.8 (2)	C14—C13—C18—C17	0.0 (3)
C7—N2—C1—C6	−138.8 (2)	N4—C13—C18—C17	179.2 (2)
N1—N2—C7—C8	−140.8 (2)	C13—C14—C15—C16	−1.6 (3)
N1—N2—C1—C2	−138.6 (2)	C14—C15—C16—C17	1.2 (4)
N1—N2—C7—C12	38.5 (3)	C15—C16—C17—C18	−0.1 (4)
C7—N2—C1—C2	39.8 (3)	C16—C17—C18—C13	−0.5 (4)
C1—N2—C7—C8	40.8 (3)	C20—C19—C24—C23	0.8 (3)
N1—N2—C1—C6	42.8 (3)	C24—C19—C20—C21	0.3 (3)
C1—N2—C7—C12	−139.9 (2)	N4—C19—C24—C23	178.77 (18)
N3—N4—C13—C18	37.7 (3)	N4—C19—C20—C21	−177.72 (18)
C13—N4—C19—C20	−166.35 (18)	C19—C20—C21—C22	−1.1 (3)
N3—N4—C19—C24	−149.82 (19)	C20—C21—C22—C23	0.8 (3)
N3—N4—C19—C20	28.2 (3)	C21—C22—C23—C24	0.3 (3)
C13—N4—C19—C24	15.6 (3)	C22—C23—C24—C19	−1.1 (3)
C19—N4—C13—C14	51.2 (3)	O1—C26—C27—C28	−1.1 (4)
N3—N4—C13—C14	−143.23 (19)	C25—C26—C27—C28	178.4 (2)
C19—N4—C13—C18	−128.0 (2)	C26—C27—C28—C29	176.5 (2)
N2—C1—C6—C5	179.3 (2)	C26—C27—C28—O2	−3.5 (4)

### Hydrogen-bond geometry (Å, °)

**Table:**

*D*—H···*A*	*D*—H	H···*A*	*D*···*A*	*D*—H···*A*
C6—H6···Br2	0.95	2.92	3.712 (3)	142
C20—H20···Br2	0.95	2.85	3.788 (2)	168
C23—H23···O2^i^	0.95	2.49	3.392 (2)	158

Symmetry codes: (i) −*x*+1, −*y*, −*z*.
